# Unique Roles of β-Arrestin in GPCR Trafficking Revealed by Photoinducible Dimerizers

**DOI:** 10.1038/s41598-017-19130-y

**Published:** 2018-01-12

**Authors:** Osamu Takenouchi, Hideaki Yoshimura, Takeaki Ozawa

**Affiliations:** Department of Chemistry, Graduate School of Science, The University of Tokyo, 7-3-1 Hongo, Bunkyo-ku, Tokyo 113-0033 Japan

## Abstract

Intracellular trafficking of G protein-coupled receptors (GPCRs) controls their localization and degradation, which affects a cell’s ability to adapt to extracellular stimuli. Although the perturbation of trafficking induces important diseases, these trafficking mechanisms are poorly understood. Herein, we demonstrate an optogenetic method using an optical dimerizer, cryptochrome (CRY) and its partner protein (CIB), to analyze the trafficking mechanisms of GPCRs and their regulatory proteins. Temporally controlling the interaction between β-arrestin and β2-adrenergic receptor (ADRB2) reveals that the duration of the β-arrestin-ADRB2 interaction determines the trafficking pathway of ADRB2. Remarkably, the phosphorylation of ADRB2 by G protein-coupled receptor kinases is unnecessary to trigger clathrin-mediated endocytosis, and β-arrestin interacting with unphosphorylated ADRB2 fails to activate mitogen-activated protein kinase (MAPK) signaling, in contrast to the ADRB2 agonist isoproterenol. Temporal control of β-arrestin-GPCR interactions will enable the investigation of the unique roles of β-arrestin and the mechanism by which it regulates β-arrestin-specific trafficking pathways of different GPCRs.

## Introduction

The localization and degradation of membrane proteins are regulated by intracellular trafficking^1,2^. The precise regulation of membrane proteins stimulated by membrane receptors is important for cells to adapt to a wide variety of extracellular stimuli. The perturbation of trafficking systems induces important diseases, including diabetes, cancers and neurodegenerative diseases^3–6^. Investigating the trafficking mechanism is therefore important for drug development and therapeutics. Intracellular trafficking is regulated by many proteins. β-Arrestin is a representative of these proteins and regulates the localization of membrane receptors, such as G protein-coupled receptors (GPCRs)^7,8^, growth factor receptors (transforming growth factor receptor (TGFR) and insulin-like growth factor receptor (IGFR))^9,10^. β-Arrestin is recruited to ligand-bound GPCRs and sequestrates the receptor from the cell surface by endocytosis. Imaging of GPCRs and β-arrestin revealed that GPCRs that stably interact with β-arrestin are retained in the cytosolic compartment, whereas GPCRs that immediately dissociate from β-arrestin are rapidly recycled back to the cell membrane^11–13^. These results suggest a significant role for β-arrestin in the intracellular trafficking of GPCRs. In addition, GPCR-bound β-arrestin functions as a signal transducer of mitogen-activated protein kinases (MAPKs) and the Ser-Thr kinase Akt in the trafficking pathway^14^. Despite the significance of β-arrestin-related trafficking, the manner by which β-arrestin switches the trafficking pathways of GPCRs in living cells is unclear.

Optogenetics, in combination with fluorescence and bioluminescence imaging techniques, is a useful technique for the spatiotemporal analysis of intracellular signaling. Photodimerizing proteins, one of many photoreceptor derived tools, are often used to control the interactions of specific proteins in living cells^15–17^. When the cryptochrome (CRY) derived from *Arabidopsis thaliana* absorbs blue light, it interacts with cryptochrome-interacting basic-helix-loop-helix 1 (CIB)^18,19^. An attractive feature of this system is that CRY and CIB reversibly interact and rapidly associate and dissociate. Herein, we demonstrate an optogenetic approach using the reversible interaction between CRY and CIB to investigate the significance of the interaction of β-arrestin with GPCRs in intracellular trafficking in living cells. Our method reveals that the light-induced interaction of β-arrestin with the β2-adrenergic receptor (ADRB2) is sufficient to trigger endocytosis of ADRB2 without ADRB2 being phosphorylated. We also clarify that the dissociation of β-arrestin promotes the recycling of ADRB2 to the plasma membrane, whereas prolonged interaction of β-arrestin with ADRB2 directs ADRB2 to the lysosomal pathway. These results demonstrate the significant role of the duration of the β-arrestin-ADRB2 interaction in sorting between intracellular trafficking pathways in living cells.

## Result

### Basic Strategy for Light-Induced Interactions between ADRB2 and β-Arrestin

**Figure 1:**
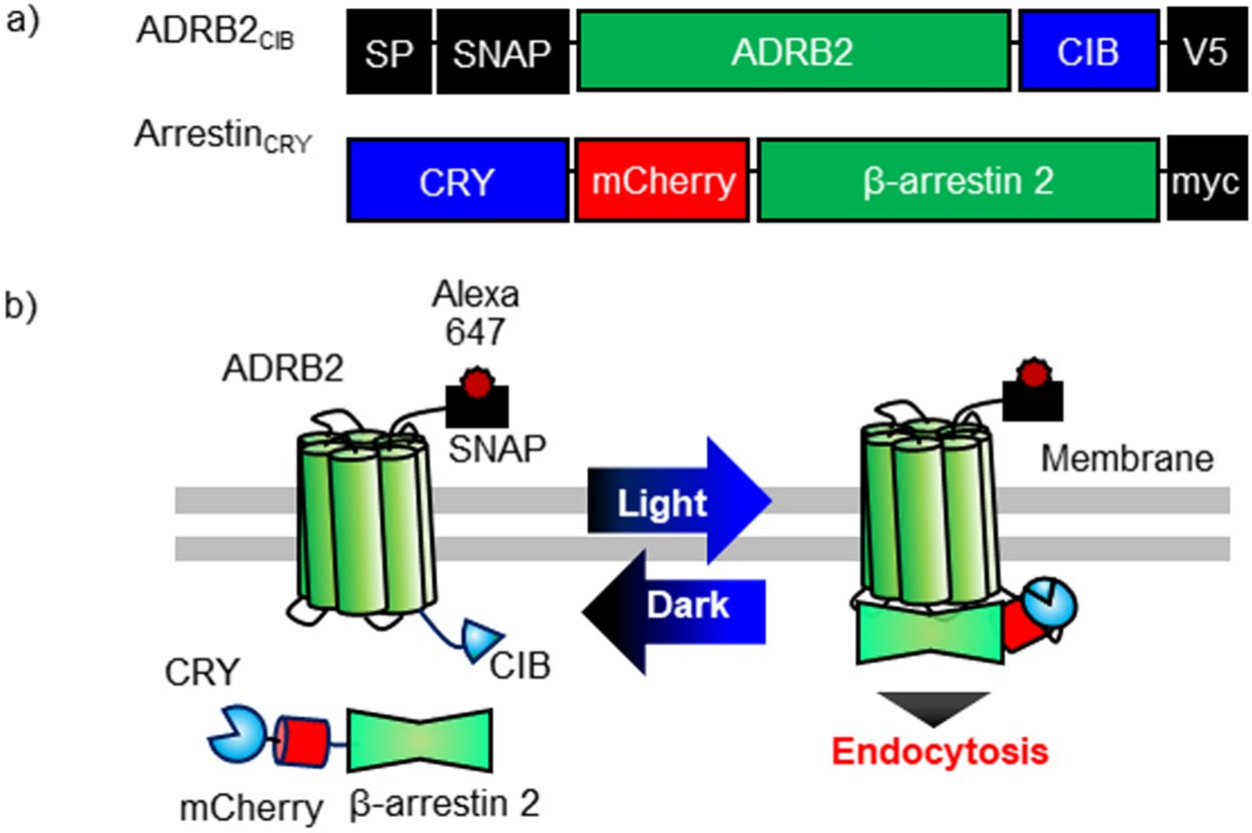
Light-based manipulation of reversible interaction between β-arrestin and ADRB2 using photodimerizers. (**a**) Schematic structures of the optogenetic manipulation system for the reversible interaction of β-arrestin with ADRB2. ADRB2_CIB_ is composed of a signal peptide (SP) derived from 5HT3A serotonin receptor that targets the protein to the cell membrane, a SNAP-tag, a β2-adrenergic receptor (ADRB2), a CIB, and a V5 epitope tag. Arrestin_CRY_ consists of a cryptochrome (CRY), an mCherry, a β-arrestin2, and a myc epitope tag. (**b**) Basic principle of light-induced interaction of β-arrestin2 with ADRB2. Before light stimulation, ADRB2_CIB_ localizes on the cell membrane, and Arrestin_CRY_ distributes throughout the cytosol. Upon stimulation with blue light, Arrestin_CRY_ interacts with ADRB2_CIB_, which triggers the endocytosis of the ADRB2_CIB_-Arrestin_CRY_ complex. Under dark conditions, Arrestin_CRY_ dissociates from ADRB2_CIB_ and redistributes into the cytosol.

ADRB2 is a GPCR that regulates cardiovascular and pulmonary functions^20,21^. Isoproterenol chloride (ISO), a specific ligand of ADRB2, activates G protein-mediated signals and induces clathrin-mediated endocytosis of ADRB2 through its interaction with β-arrestin. To temporally control the interaction between ADRB2 and β-arrestin by external blue light, we connected SNAP-tag-fused ADRB2 to the N-terminus of CIB (named ADRB2_CIB_); in addition, mCherry-fused β-arrestin 2 was attached to the C-terminus of CRY (denoted Arrestin_CRY_) (Fig. 1a). Each domain was connected to flexible linkers composed of glycine and serine to allow the dynamic motion of the fusion proteins. Before blue light irradiation, ADRB2_CIB_ locates on the cell membrane, and Arrestin_CRY_ distributes throughout the cytosol (Fig. 1b). Stimulation with blue light induces translocation of Arrestin_CRY_ to ADRB2_CIB_ on the cell membrane by the interaction between CRY and CIB, thereby triggering endocytosis of ADRB2_CIB_-Arrestin_CRY_ complexes. After irradiation is stopped, Arrestin_CRY_ is redistributed in the cytosol by the dissociation of CRY and CIB.

### Light-Induced Endocytosis of ADRB2_CIB_

**Figure 2:**
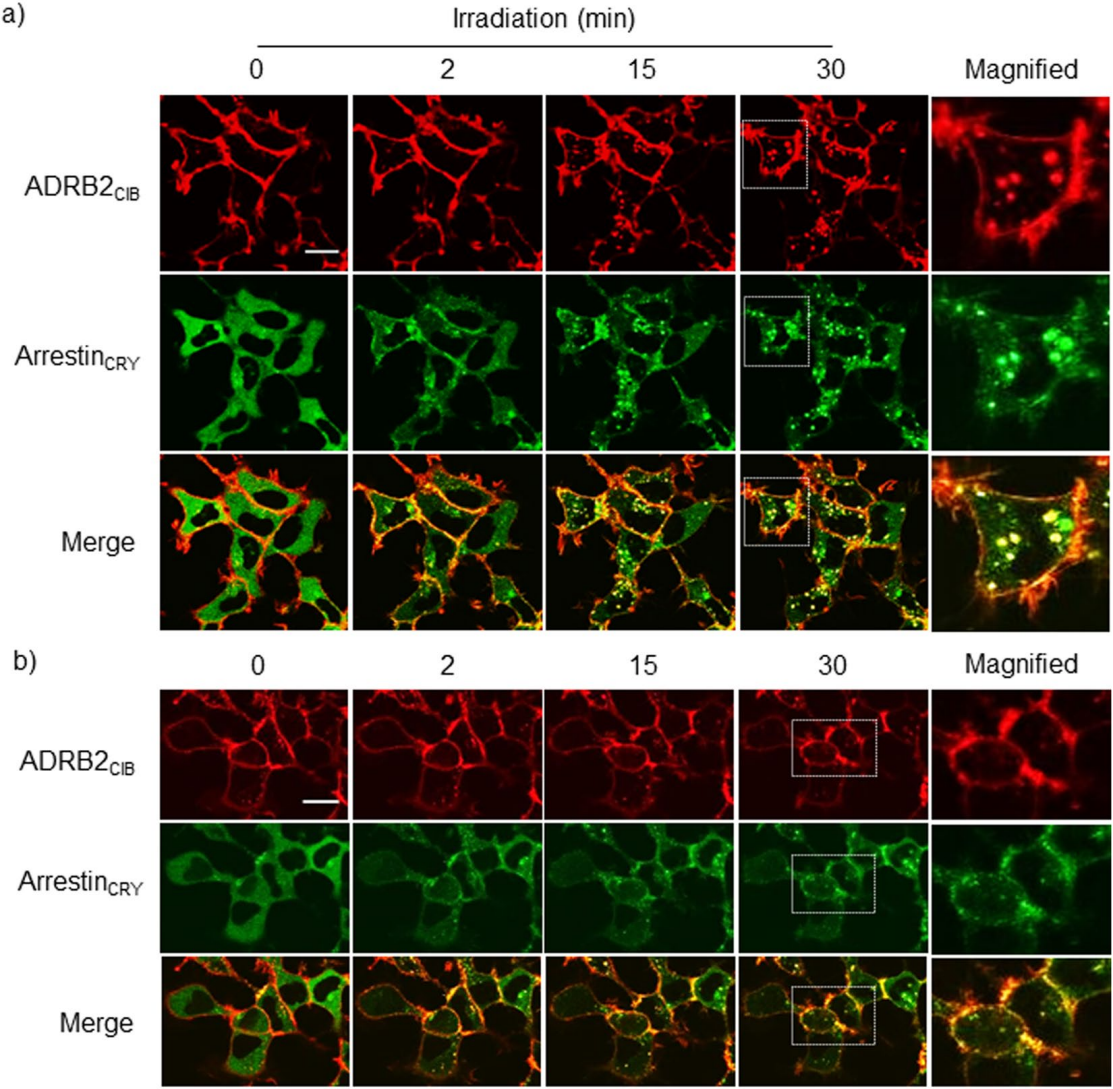
Light-induced endocytosis of ADRB2_CIB_. Images of ADRB2_CIB_ and Arrestin_CRY_ fluorescence in HEK293_opt_ cells irradiated with blue light for 0, 2, 15 and 30 min under a confocal microscope (**a**), and images of these cells pretreated with 30 μM Dyngo4a for 30 min and then irradiated by blue light for 0, 2, 15 and 30 min (**b**). Red, ADRB2_CIB_; green, Arrestin_CRY_. Scale bar, 20 μm.

To determine whether light irradiation controls the interaction between ADRB2 and β-arrestin, HEK293 cells stably expressing ADRB2_CIB_ and Arrestin_CRY_ (HEK293_opt_) were irradiated with blue light under a confocal fluorescence microscope (Fig. 2a, Movie S1). Arrestin_CRY_ was translocated from the cytosol to the cell membrane a few min after the start of irradiation and then moved back into the cytosol to form a dot-like structure. ADRB2_CIB_ fluorescent spots appeared in the cytosol at 15 min, and their number gradually increased until 30 min of irradiation. The fluorescent spots mostly colocalized with Arrestin_CRY_. In a control experiment, a fusion protein comprising SNAP-tag and ADRB2 without CIB was expressed in HEK293 cells. The localization of Arrestin_CRY_ was unchanged upon stimulation with blue light due to the lack of the CIB portion (Supplementary Fig. 1a). Furthermore, fluorescent spots in the HEK293 cells expressing Arrestin_CRY_ and cell membrane-anchored CIB (Myr-Venus-CIB) were not observed during irradiation, despite Arrestin_CRY_ being translocated to the cell membrane. To analyze the results quantitatively, we counted the number of spots containing ADRB2_CIB_, ADRB2, and Myr-Venus-CIB in the obtained images (Supplementary Fig. 1b). A significant increase in the number of spots was observed after light irradiation in case of ADRB2_CIB_. In contrast, the number of spots containing ADRB2 or Myr-Venus-CIB did not change after light stimulation. The results indicate that recruitment of β-arrestin to ADRB2 is necessary for appearance of the spots. In addition, the cells expressing Myr-Venus-CIB and Arrestin_CRY_ were irradiated for 120 min (Supplementary Fig. 1c). The number of spots containing Myr-Venus-CIB did not increase under the prolonged irradiation.

We next examined the endocytosis of ADRB2 after ADRB2_CIB_-Arrestin_CRY_ interaction. ADRB2 is endocytosed with β-arrestin in a clathrin-mediated manner^22^. Segregation of clathrin-coated pits from the cell membrane is regulated by dynamin GTPases. To show that the fluorescent spots resulted from clathrin-mediated endocytosis, we pretreated the HEK293_opt_ cells with a dynamin inhibitor, Dyngo4a. The fluorescent spots did not appear in the presence of Dyngo4a after the stimulation by light (Fig. 2b). To quantitate this inhibitory effect, we quantified the number of ADRB2_CIB_ fluorescent spots and the colocalization of Arrestin_CRY_ with ADRB2_CIB_ using the quantitative parameter Manders’ colocalization coefficient, which is proportional to the total amount of fluorescence intensity from Arrestin_CRY_ in the pixels where it colocalizes with ADRB2_CIB_ (Supplementary Fig. 2a,b). The number of spots increased with prolonged irradiation time in the absence of Dyngo4a. In contrast, light irradiation did not induce an increase in the number of spots in the presence of Dyngo4a although the colocalization of Arrestin_CRY_ with ADRB2_CIB_ was induced. Considering these results, we concluded that the endocytosis of ADRB2_CIB_ was triggered by light-induced interaction of β-arrestin with ADRB2, and the observed ADRB2_CIB_ fluorescent spots were ADRB2_CIB_-containing vesicles produced by clathrin-mediated endocytosis.

We further investigated the time dependency of the light-induced endocytosis of ADRB2_CIB_. After HEK293_opt_ cells were stimulated with blue light for different durations, the amount of ADRB2_CIB_ on the cell surface was quantified using an ELISA^23^ (Supplementary Fig. 3a). The amount of ADRB2_CIB_ on the cell surface decreased with increasing irradiation time. We also examined the light intensity dependency of endocytosis (Supplementary Fig. 3b). Endocytosis was promoted by increased light intensity and plateaued at 3 mW/cm^2^. The amount of ADRB2_CIB_ endocytosed by 3 mW/cm^2^ light was more than that induced by 0.01 μM ISO and less than that induced by 0.1 and 1.0 μM ISO. These results demonstrate that the endocytosis of ADRB2_CIB_ can be controlled by modulating light intensity and irradiation time.

### Localization of ADRB2_CIB_ after Light-Induced Endocytosis

Most GPCRs are transported to an endocytic pathway after internalization. To confirm that ADRB2_CIB_ is transported to endosomes after light-induced endocytosis, we immunostained early and late endosomes using antibodies specific to endosome marker proteins Rab5 and Rab7, respectively (Supplementary Fig. 4a,b). The internalized ADRB2_CIB_ was partially localized in Rab5- or Rab7-positive vesicles after 30 min of irradiation. The residual ADRB2_CIB_ did not colocalize with the endosomes, indicative of sorting to other endosomes or lysosomes. Based on these results, we concluded that part of the ADRB2_CIB_ is transported to endosomes after light-induced endocytosis.

### Induction of the Recycling of ADRB2_CIB_ in Dark Conditions

**Figure 3:**
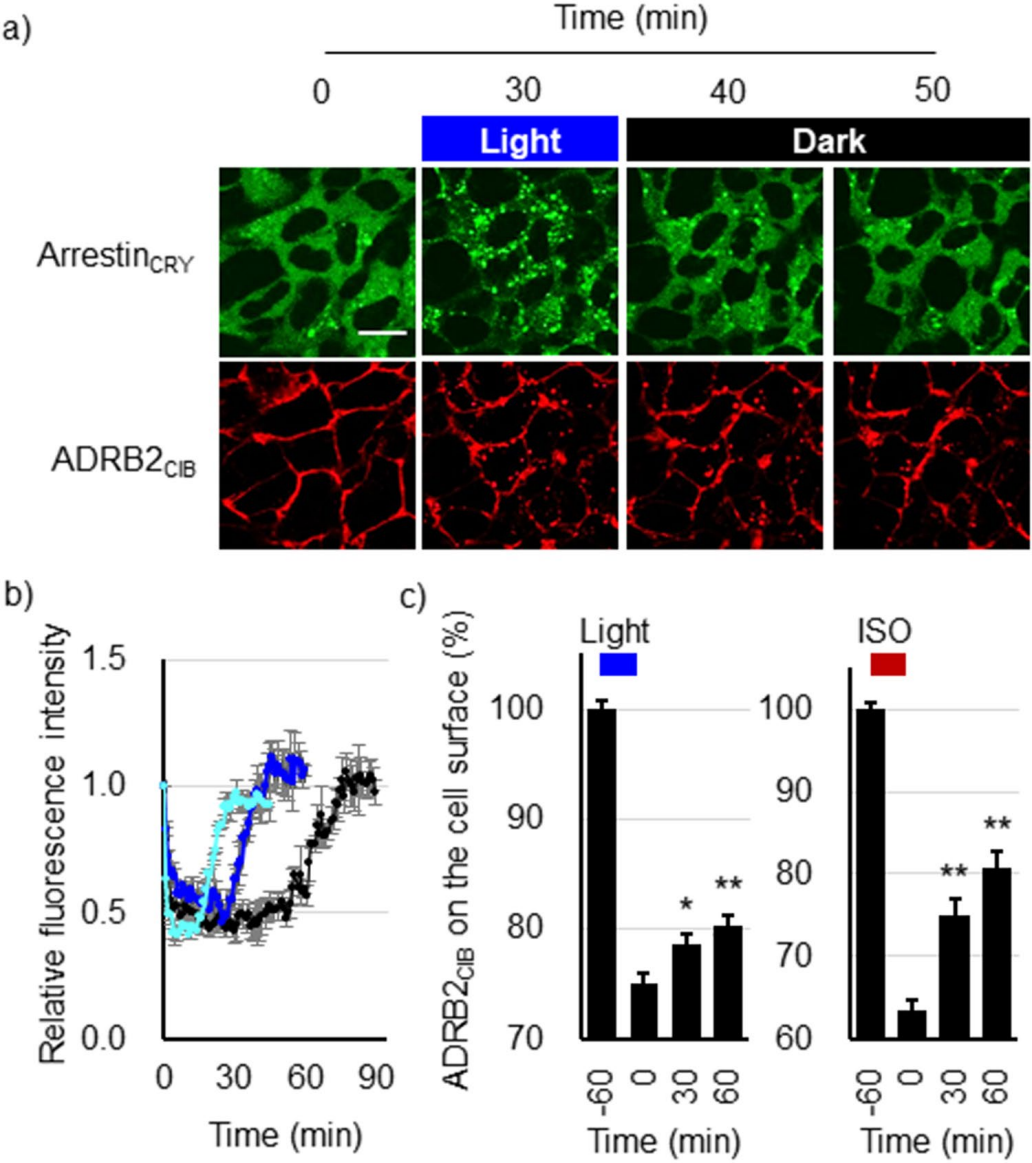
Induction of the recycling pathway in the dark after light irradiation. (**a**) Images of Arrestin_CRY_ and ADRB2_CIB_ fluorescence in HEK293_opt_ cells stimulated with blue light for 30 min and then incubated for 10 and 20 min (total of 40 and 50 min observation, respectively) without blue light irradiation. Red, ADRB2_CIB_; green, Arrestin_CRY_. Scale bar, 20 μm. (**b**) Time courses of the fluorescence intensity of cytosolic Arrestin_CRY_ in each cell. The cells were stimulated with blue light for 15 min (pale blue), 30 min (deep blue), and 60 min (black) and then incubated in the dark. Each time course was normalized to the fluorescence intensity at 0 min. Bars: mean ± s.e.m (n = 9 from three individual experiments). (**c**) Recovery of ADRB2_CIB_ on the cell surface after stopping irradiation. HEK293_opt_ cells were stimulated with blue light (left) or 1.0 μM isoproterenol (right) for 60 min. The cells were then incubated at 37 °C in the dark for the indicated times. The amount of ADRB2_CIB_ on the cell surface was quantified using an ELISA. Bars: mean ± s.e.m (n = 12 from three individual experiments). The statistical analysis was performed with Bonferroni post-hoc test (0 min v.s. 30 or 60 min) *P values < 0.05 (*P* = 0.024), **P values < 0.01 (Light; *P* = 1.9 × 10^−3^, ISO; *P* = 8.2 × 10^−5^ (30 min), 6.6 × 10^−7^ (60 min)).

To show the reversibility of the light-induced interaction between ADRB2_CIB_ and Arrestin_CRY_, we irradiated the HEK293_opt_ cells with blue light for 30 min and then incubated them in the dark. After Arrestin_CRY_ colocalized with ADRB2_CIB_, Arrestin_CRY_ redistributed uniformly in the cytosol under dark conditions (Fig. 3a). The fluorescence intensity of Arrestin_CRY_ in the cytosol decreased 1 min after irradiation was started and then recovered to the basal level 10 min after irradiation ceased (Fig. 3b). The colocalization coefficient of Arrestin_CRY_ with ADRB2_CIB_ decreased after stopping irradiation (Supplementary Fig. 5). The results confirm that Arrestin_CRY_ dissociated from ADRB2_CIB_ in the dark.

To demonstrate the recycling of ADRB2_CIB_ to the cell surface after the dissociation of Arrestin_CRY_, we quantified the amount of ADRB2_CIB_ on the cell surface using an ELISA after stopping irradiation. The HEK293_opt_ cells were stimulated with blue light for 60 min and then incubated in the dark. The amount of ADRB2_CIB_ on the cell surface decreased to 75% after light stimulation and recovered to 80% after 60 min of incubation in the dark (Fig. 3c, left). This result indicates that the recycling of the ADRB2_CIB_ was triggered by the dissociation of β-arrestin from ADRB2 in the dark. Furthermore, in HEK293_opt_ cells stimulated with ISO, the amount of ADRB2_CIB_ on the cell surface recovered from 63 to 77% after ISO was eliminated (Fig. 3c, right) The temporal changes in recycling after light irradiation ceased were similar to those in recycling when ISO was eliminated, demonstrating that temporal control of the dissociation between β-arrestin and ADRB2 is useful for investigating the intracellular dynamics of ADRB2 and regulatory proteins in the recycling pathway.

### Sorting ADRB2_CIB_ into the Lysosome Pathway after Prolonged Light Irradiation

**Figure 4:**
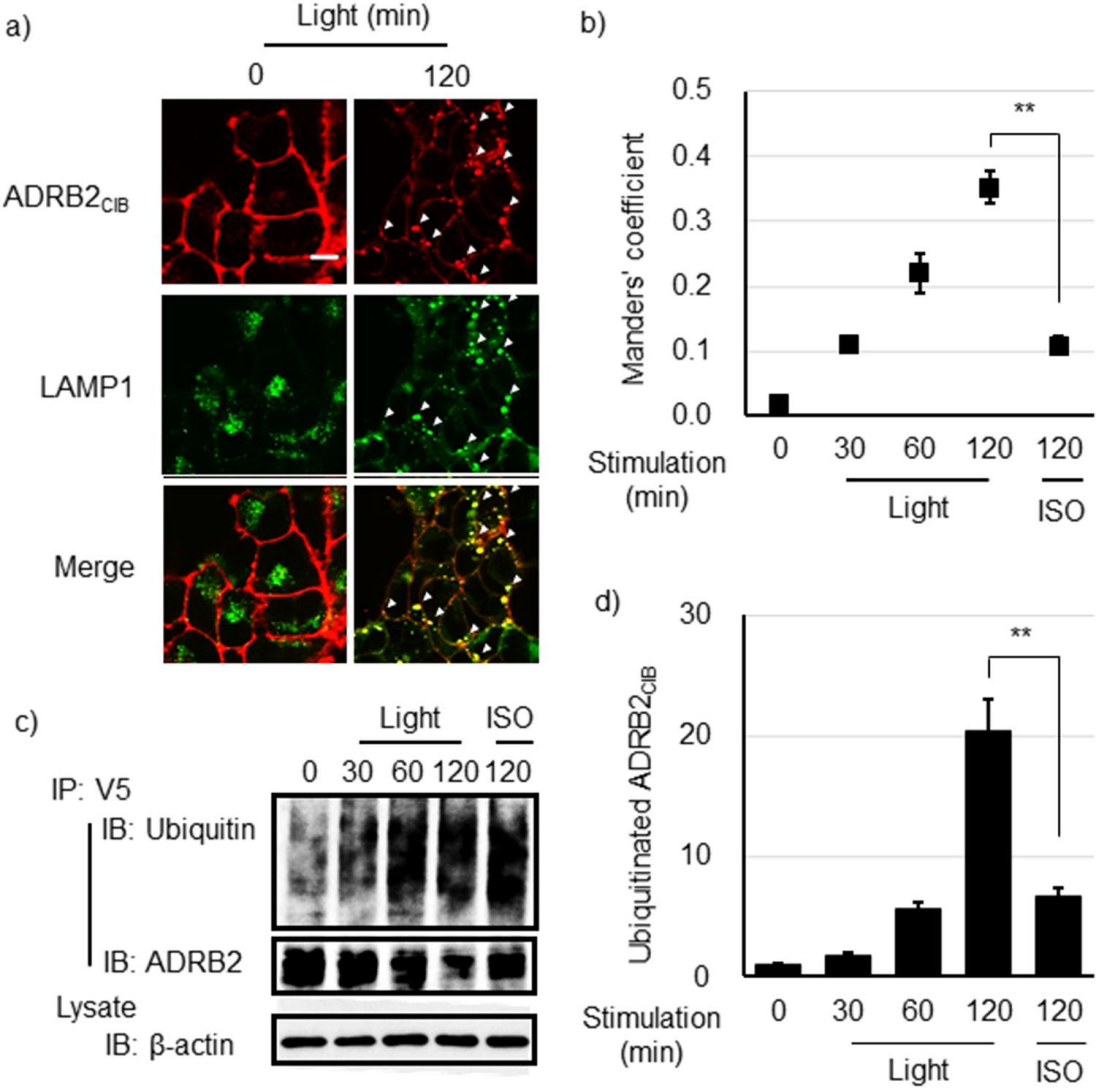
Lysosome sorting of ADRB2_CIB_ after prolonged irradiation. (**a**) Images of ADRB2_CIB_ and lysosome marker LAMP1 fluorescence in HEK293_opt_ cells before and after blue light irradiation for 120 min. The white arrowheads indicate the colocalization of ADRB2_CIB_ and LAMP1. Red, ADRB2_CIB_; green, LAMP1. Bar, 20 μm. (**b**) Temporal changes in the colocalization of ADRB2_CIB_ with lysosomes in HEK293_opt_ cells stimulated with blue light for the indicated time or with ISO for 120 min. Data are shown as the mean ± s.e.m. (n = 9 from two independent experiments) The statistical analysis was performed unpaired Student’s t-test (two-tailed). **P < 0.01 (*P* = 2.0 × 10^−7^). (**c**) IP-Western blotting analysis for the detection of ubiquitinated ADRB2_CIB_. HEK293_opt_ cells were stimulated with blue light for the indicated time or with 1.0 μM ISO for 120 min. ADRB2_CIB_ was immunoprecipitated using an anti-V5-tag antibody. Ubiquitin, ADRB2 and β-actin were blotted with antibodies specific to them. (**d**) The quantified temporal changes in ubiquitinated ADRB2_CIB_ were calculated as the ratio of ubiquitinated ADRB2_CIB_ to total ADRB2_CIB_ quantities, which were determined in the Western blot analysis. The ratio values were normalized to their value at 0 min. Bars: mean ± s.e.m. (n = 9 from three independent experiments). The statistical analysis was performed unpaired Student’s t-test (two-tailed). **P < 0.01 (*P* = 1.1 × 10^−4^).

We next investigated the effects of a prolonged interaction of Arrestin_CRY_ with ADRB2_CIB_ on the sorting of ADRB2_CIB_. HEK293_opt_ cells were irradiated with blue light for 120 min. Lysosomes were immunostained with an antibody specific to the lysosome marker protein LAMP1 (Fig. 4a). Sustained light irradiation for 120 min resulted in the disappearance of the ADRB2_CIB_ from the cell membrane; instead, ADRB2_CIB_ predominantly localized in lysosomes. Furthermore, we examined the colocalization of ADRRB2_CIB_ with lysosomes (Fig. 4b). The Manders’ coefficient increased with increased irradiation time, indicating that the prolonged interaction of Arrestin_CRY_ with ADRB2_CIB_ drives ADRB2_CIB_ to lysosomes. We also examined the ubiquitination of ADRB2_CIB_, which is a crucial signal that sorts GPCRs to lysosomes^24^. Furthermore, ADRB2_CIB_ was ubiquitinated to a greater degree when the irradiation time was increased to 120 min (Fig. 4c,d). These results demonstrate that prolonged interaction of β-arrestin with ADRB2, stimulated by blue light, promotes the sorting of ADRB2_CIB_ to lysosomes.

### Monitoring a Protein that Regulates ADRB2-β-arrestin Complexes

**Figure 5:**
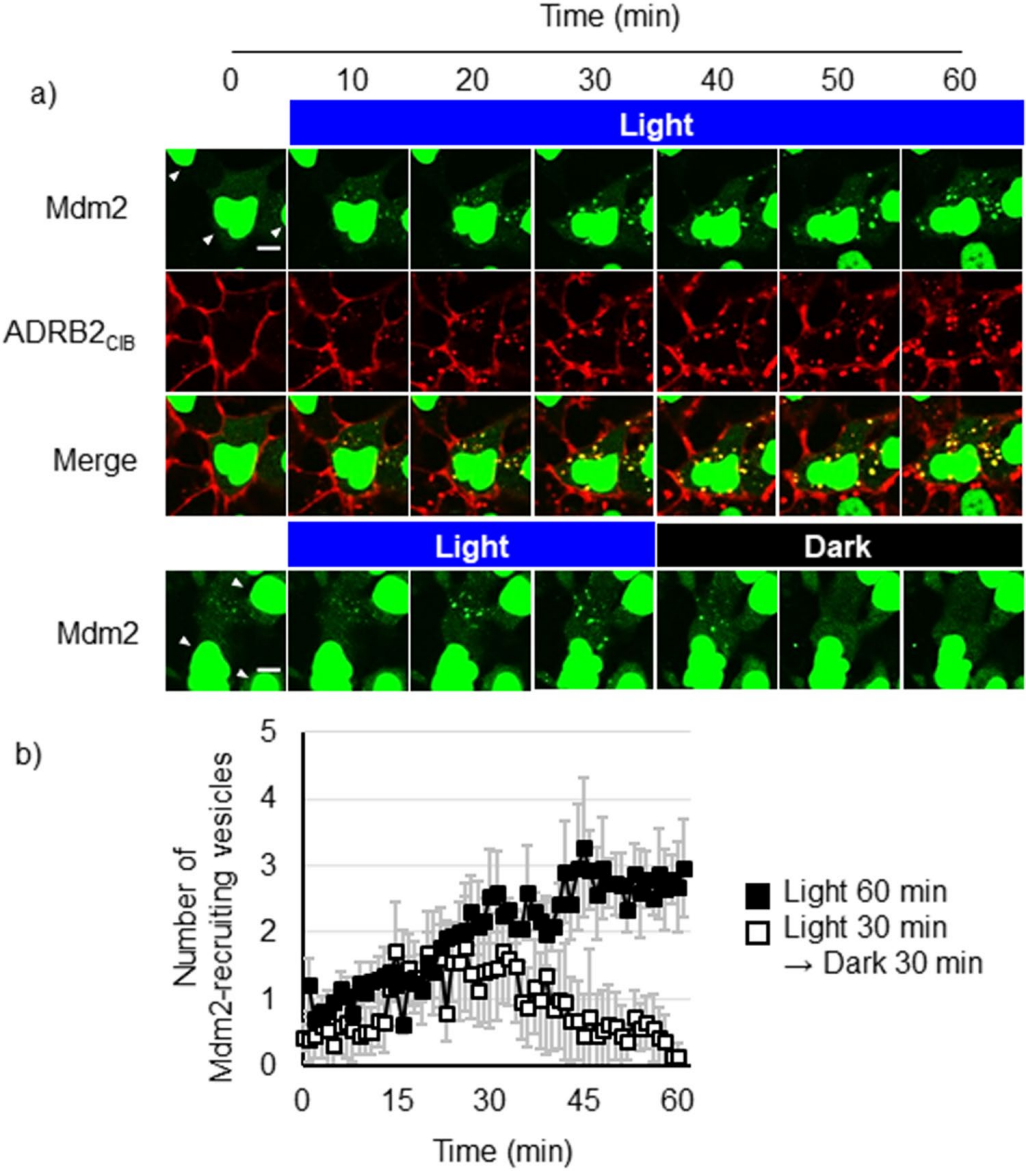
Observation of the dynamics of Mdm2 and ADRB2_CIB_. (**a**) Time-lapse images of Mdm2 and ADRB2_CIB_ in HEK293_optCLIP_ cells. Cells expressing mCherry-fused Mdm2 were stimulated with blue light for 60 min (upper) or for 30 min followed by an additional 30 min in the dark (lower). Red, ADRB2_CIB_; green, mCherry-fused Mdm2. Arrowheads, nuclei. Bar, 10 μm. (**b**) Temporal changes in the number of Mdm2-recruiting vesicles per cell. The filled squares show the number of vesicles in cells irradiated for 60 min. The open squares show the number of the vesicles in cells irradiated for 30 min and then incubated in dark for 30 min. Error bars: mean ± s.e.m. (n = 14 cells from three individual experiments).

Some regulatory proteins are recruited to ADRB2-β-arrestin complexes; monitoring these regulatory proteins is important to determine their dynamics and functions in trafficking pathways. Mdm2 is a protein that regulates the endocytosis of ADRB2^25^. Although recent studies suggested that Mdm2 has several important roles in the regulation of other membrane receptors, such as the induction of endocytosis of opioid receptors and the activation of a signaling pathway regulated by an IGFR, the dynamics for Mdm2 recruitment to these receptors is unclear^26,27^. To monitor the intracellular dynamics of Mdm2, we established a cell line that stably expresses ADRB2_CIB_ and Arrestin_CRY_, but we replaced mCherry with a CLIP tag (HEK293_optCLIP_). HEK293_optCLIP_ cells were transfected with cDNA coding mCherry-fused Mdm2 and were then stimulated with blue light under a confocal microscope (Fig. 5a). Before stimulation, most Mdm2 localized on the nucleus, and the residual Mdm2 distributed throughout the cytosol. Several fluorescent spots of Mdm2 were observed in the cytosol after 15 min of light irradiation, and they colocalized with the ADRB2_CIB_-containing vesicles. This result suggests that the cytosolic Mdm2 is recruited to the light-induced ADRB2-β-arrestin complexes. The colocalization of Mdm2 with ADRB2_CIB_-containing vesicles was sustained during continuous irradiation (Fig. 5a upper, Movie S2a). In contrast, the Mdm2 dissociated from ADRB2_CIB_ 10 min after irradiation ceased (Fig. 5a lower, Movie S2b). We counted the number of Mdm2-recruiting vesicles in the cytosol (Fig. 5b). The number of Mdm2-recruiting vesicles increased with increasing irradiation time but decreased with increasing incubation in the dark. These results indicate that the interaction of β-arrestin with ADRB2 promotes the recruitment of Mdm2 to the ADRB2-β-arrestin complex and that the interaction of Mdm2 with the ADRB2_CIB_ complex continues until Arrestin_CRY_ dissociates from ADRB2_CIB_.

### Examination of the Ability of Arrestin_CRY_ to Desensitize ISO-activated ADRB2_CIB_

Desensitization of ligand-activated GPCRs is one of the important functions of β-arrestin. To examine whether light-induced recruitment of Arrestin_CRY_ desensitizes the ISO-activated ADRB2_CIB_, we investigated cAMP production after ISO stimulation under blue light irradiation using cAMP biosensor (Supplementary Fig. 6). The light irradiation did not affect luminescence intensity changes, suggesting that light-induced recruitment of Arrestin_CRY_ does not promote desensitization of ADRB2_CIB_.

### Investigation of ADRB2_CIB_ Activity during Light Stimulation

To confirm the activity state of ADRB2_CIB_ during the light stimulation, HEK293_opt_ cells were transfected with cDNA coding a GFP-fused nanobody (Nb80), which binds specifically to the active state of ADRB2 interacting to G proteins^28^. The cells were stimulated with light or isoproterenol for 30 min under a confocal microscope. The GFP-Nb80 colocalized with ADRB2_CIB_-containing endosomes after ISO stimulation, indicating that the ADRB2_CIB_ has an ability to activate the downstream signaling upon ISO stimulation (Supplementary Fig. 7a). In contrast, the colocalization of GFP-Nb80 with ADRB2_CIB_ was not observed after light stimulation. To demonstrate the results quantitatively, we quantified the number of spots containing GFP-Nb80 and the colocalization of GFP-Nb80 with ADRB2_CIB_. The spots containing GFP-Nb80 were not observed 30 min after light irradiation, although ISO stimulation induced increases in the number of spots (Supplementary Fig. 7b). The Manders’ colocalization coefficient did not change after light stimulation, but increased after ISO stimulation (Supplementary Fig. 7c). These results suggest that endocytosed ADRB2_CIB_ by light stimulation does not form the active state required for G protein signaling.

### Investigating the Ability of Arrestin_CRY_ to Induce Phosphorylation of ERK1/2

β-Arrestin, after interacting with ADRB2, functions as a scaffold for the activation of MAPK signaling^29^. Ligand stimulation induces phosphorylation of ADRB2 via G protein-coupled receptor kinases. Phosphorylation of ADRB2 increases the affinity of ADRB2 for β-arrestin and changes the conformation of β-arrestin to form a complex with signaling molecules such as MAPK/ERK kinase (MEK) and ERK1/2, which are then phosphorylated through the interaction with β-arrestin^30–32^. To examine whether Arrestin_CRY_ induces phosphorylation of ERK1/2 after the light-induced interaction of Arrestin_CRY_ with ADRB2_CIB_, HEK293_opt_ cells were irradiated with blue light for 60 min. The phosphorylated ERK1/2 level was evaluated with its specific antibody (Supplementary Fig. 8a). ISO stimulation increased the amount of phosphorylated ERK1/2, whereas the phosphorylated ERK1/2 level decreased after light stimulation. To clarify the reason why light stimulation failed to increase the phosphorylated ERK1/2 level, we investigated phosphorylated ADRB2 (Supplementary Fig. 8b). The phosphorylation of ADRB2_CIB_ was not detected after light irradiation. Considering that the phosphorylation of ADRB2 triggers a conformational change in β-arrestin that enables the recruitment of ERK1/2 and MEK to β-arrestin^30–32^, we concluded that Arrestin_CRY_ does not recruit ERK1/2 or MEK to the Arrestin_CRY_-ADRB2_CIB_ complex due to the lack of ADRB2 phosphorylation.

### Applicability of the Light Activation System to Other Membrane Receptors

To investigate the applicability of the present system to other membrane receptors, we replaced ADRB2 with different GPCRs such as the neurotensin receptor, muscarinic acetylcholine receptor M3, corticotropin releasing-factor receptor, and vasopressin 2 receptor. All the GPCRs that were investigated in this study localized on the cell surface before irradiation with blue light. Light stimulation induced translocation of Arrestin_CRY_ to the cell surface and endocytosis of the GPCRs (Supplementary Fig. 9a). Furthermore, we applied the method to a non-GPCR receptor, transforming growth factor 3 receptor (TGF3R). TGF3R localized on the cell surface before light stimulation, whereas light stimulation led to the endocytosis of TGF3R, indicating that endocytosis of TGF3R is controllable by the recruitment of β-arrestin to TGF3R. These results demonstrate that the present method is widely applicable to inducing the endocytosis of membrane receptors in living cells. A previous report demonstrated that ERK1/2 phosphorylation was promoted by chemical dimerizer-induced interaction of β-arrestin with V2R^23^, implying different regulations of MAPK activation between ADRB2 and V2R. To examine the difference, we quantified phosphorylated ERK1/2 after light-induced interaction of Arretin_CRY_ with V2R_CIB_. In contrast to the case of ADRB2, the light-induced interaction of β-arrestin with V2R promoted phosphorylation of ERK1/2 30 min after light irradiation (Supplementary Fig. 9b). The results suggest that β-arrestin regulates ERK1/2 phosphorylation in different manners depending on the GPCRs.

## Discussion

We developed an optogenetic method for the analysis of intracellular trafficking of ADRB2 regulated by β-arrestin. We clarified the significance of the interaction of β-arrestin in the intracellular trafficking of ADRB2 using the CRY-CIB system. Endocytosis was promoted by increasing the time that ADRB2 interacts with β-arrestin. The ADRB2 on the cell surface was recovered after β-arrestin dissociated from ADRB2 in the dark, indicating that the recycling pathway was triggered by the dissociation of β-arrestin from ADRB2. Furthermore, ubiquitination and lysosome sorting were accelerated by prolonged irradiation. These results indicate that the duration of β-arrestin interaction with ADRB2 determines whether ADRB2 takes the lysosomal pathway. In addition, we monitored Mdm2 binding to and dissociating from ADRB2_CIB_-Arrestin_CRY_ complexes using light irradiation. Our method monitors the dynamics of ADRB2 and its regulatory proteins in the trafficking pathways. Light-induced manipulation of the interaction of β-arrestin with ADRB2 consequently clarified the regulation of the intracellular trafficking of ADRB2. The present system will be a robust tool for investigating the dynamics of a wide variety of GPCRs and their regulatory proteins in trafficking pathways. Furthermore, we examined other functions of β-arrestin such as desensitization and signaling. Light-induced interaction of β-arrestin with ADRB2 did not promote degradation of cAMP or activation of MAPK. The results indicate that the present method is applicable to controlling the trafficking of ADRB2 without inducing the desensitization or signaling, which is beneficial for investigation of mechanisms of the β-arrestin-mediated trafficking of ADRB2 in living cells and animals.

Controlling the interaction of GPCRs with β-arrestin using the CRY-CIB photoactivation system has several advantages. Previous methods to control the interaction of GPCRs with β-arrestin were based on a chemical approach^23,33^. A pair of chemical dimerizers (FKBP-FRB) were used to control the interaction of β-arrestin with vasopressin receptors or chemokine receptors. However, it is difficult to eliminate rapamycin by simple washing procedures due to its high affinity for FKBP^34^, suggesting that the chemical approach is not suitable for the temporal manipulation of these interactions. The CRY-CIB system is a robust tool to temporally control the reversible interaction of β-arrestin with membrane receptors. The other advantage of the present method is that it can be applied to a wide variety of membrane receptors by connecting CIB to the C-terminus of the target membrane receptor. This approach is useful for the manipulation of orphan GPCRs, whose ligands are undetermined. Recent studies have demonstrated that the trafficking of an orphan GPCR contributes to the pathology of Alzheimer disease^35,36^. However, knowing its specific ligands is required for further analysis of the pathological process. The present method will be useful for the analysis of such orphan GPCRs, without requiring their specific ligands, because the trafficking of GPCRs is controllable by external light. The present method can potentially determine the effects of trafficking a wide variety of membrane receptors on important diseases.

We induced prolonged interactions of β-arrestin with ADRB2 using light irradiation. GPCRs are divided into two classes (class A and class B) depending on their affinity with β-arrestin^14^. ADRB2 is a class A GPCR, members of which interact with β-arrestin for a short period after stimulation by their ligands. We artificially prolonged the interaction of β-arrestin with ADRB2 using light stimulation. The sustained interaction led to greater ubiquitination and lysosome sorting of ADRB2 than stimulation with ISO, suggesting that the duration of the interaction between ADRB2 and β-arrestin determines the amount of ubiquitination and lysosome sorting. In addition, we demonstrated the light-induced endocytosis of 4 different types of GPCRs and a TGF3R. The light-endocytosed GPCRs are possibly transported in different kinetics because the trafficking is largely influenced by the amino acid residues in the C-terminus^12^. The investigation of the differences provides us valuable information about the significant roles of C-terminus of GPCRs in kinetics regulation of their trafficking without effects of other complicated factors such as ligand affinity to GPCRs and G protein signaling.

We examined the significance of the phosphorylation of ADRB2 in trafficking and signal induction by artificially manipulating the interaction of β-arrestin with ADRB2. The direct manipulation of interactions between specific proteins has been a robust approach for analyzing the significance of protein-protein interactions in complicated intracellular networks^15^. The ligands of ADRB2 activate G proteins via ADRB2 on the cell surface, and the G protein signaling leads to phosphorylation of ADRB2, which increases the affinity of ADRB2 for β-arrestin. The interaction of β-arrestin with ADRB2 on the cell surface induces the endocytosis of ADRB2. We artificially induced the interaction between ADRB2 and β-arrestin via the CRY-CIB system without phosphorylating ADRB2. Light stimulation triggered the endocytosis of ADRB2_CIB_, indicating that phosphorylation of ADRB2 is not necessary to initiate the recruitment of endocytic proteins such as clathrin and Mdm2 to ADRB2-β-arrestin complexes. However, the maximum induction level of the endocytosis of ADRB2_CIB_ after light stimulation was lower than that induced by 0.1 and 1.0 μM ISO. The result implies that phosphorylation of ADRB2 is required for the efficient endocytosis of ADRB2 from the plasma membrane. We also evaluated the induction level of MAPK signaling after light irradiation. The level of phosphorylated ERK1/2 unexpectedly decreased after light irradiation. Although the reason for the phosphorylation decrease after light stimulation is not clear, the result indicates that other factors increase the amount of phosphorylated ERK1/2. For example, phosphorylated ADRB2 facilitates β-arrestin adopting a specific conformation to form an ADRB2-β-arrestin-MEK-ERK1/2 complex^30–32^. We speculated that the lack of increase in phosphorylated ERK1/2 after light stimulation was due to the lack of phosphorylated ADRB2. To confirm the conformation of β-arrestin, several FRET- and BRET-based indicators were reported^32,37^. However, these indicators contain bioluminescent, fluorescent proteins or chemical dyes, of which absorption and emission wavelength overlap with absorption wavelength of CRY, suggesting a possibility that the overlap hampers a precise detection of the conformation of β-arrestin. To overcome the issue, use of a FRET indicator with another absorption and emission wavelength such as mKate and iRFP will be needed. Combination of a novel indicators and the present system will provide powerful approaches for investigation of the relevance between conformation of β-arrestin and intracellular trafficking on cell surface and endosomes. In addition, several studies have demonstrated that the rapamycin-induced interaction of β-arrestin with vasopressin receptors or chemokine receptors promotes the phosphorylation of ERK1/2^23,33^. Light-induced interaction of β-arrestin with V2R also activated the phosphorylation of ERK1/2. The result using ADRB2 is completely different from the results of the reports and our result, suggesting that the activation of MAPK signaling depends on the type of GPCR. We consequently suggested that the light-induced interaction of β-arrestin with ADRB2 is not sufficient for the activation of MAPK signaling, which is a significant clue to understanding the mechanisms of activation of β-arrestin-mediated signals.

We developed the photoinducible GPCR-β-arrestin interaction systems, and utilized them for investigation of the significant roles of β-arrestin on the endocytosis and intracellular trafficking of GPCRs. In addition, there are other potential applications of the present methods. Previous studies demonstrated importance of GPCR conformations for activation and inactivation of downstream signaling^28^. However, it is not well investigated whether the conformational state of GPCRs influences on their intracellular trafficking. The trafficking of GPCRs in a specific conformation may be possibly investigated using the present system together with antagonists or inverse agonists that induce specific GPCR conformations. This approach will clarify significant roles of conformational states of GPCRs on intracellular trafficking. In addition, a previous study also suggested the possibility of the endocytosis of G proteins with some GPCRs^38^, whereas another study on single molecule imaging reported that G protein-ADRB2 complexes did not colocalize with clathrin-coated pits^39^. Thus, it is not clear whether the interaction of G protein with GPCRs affects their endocytosis. The present system may be applicable to clarify the impact of G proteins on the receptor internalization by comparing the dynamics of endocytosis between ligand-activated GPCRs interacting with G proteins and light-stimulated GPCRs without G proteins. Furthermore, the present method has a potential to control the oligomeric state of the GPCRs. GPCRs form dimers and oligomers upon stimulation of their ligands, which is important for recruitment of their regulatory proteins on cell surface^40^. Based on the fact that the light-activated CRY and CIB forms oligomers^41^, ADRB2_CIB_ and Arrestin_CRY_ also form oligomers upon light stimulation. Light control of the oligomeric state of ligand-stimulated GPCRs will be a useful approach to investigate the oligomerization of GPCRs affecting their regulatory proteins in living cells.

In conclusion, we developed a novel method that uses the CRY-CIB system to manipulate the interaction between β-arrestin and ADRB2. Temporal manipulation of the interaction between β-arrestin and ADRB2 using this optogenetic system revealed the β-arrestin-mediated regulation of the intracellular trafficking of ADRB2. Endocytosis of ADRB2 was promoted by increased light intensity and irradiation time. The dissociation of β-arrestin from ADRB2 under dark conditions triggered the recycling of the endocytosed ADRB2 to the cell surface, whereas the prolonged interaction of β-arrestin with ADRB2 promoted the sorting of ADRB2 to lysosomes. The duration of the reversible interaction of β-arrestin with ADRB2 therefore determines the intracellular trafficking of ADRB2. In addition, the recruitment of the regulatory protein Mdm2 to ADRB2-β-arrestin complexes was driven by light induction of the interaction between β-arrestin and ADRB2. Our method will be effective for investigating the dynamics of GPCRs and their regulatory proteins in trafficking pathways. Furthermore, MAPK signaling was not activated through the light-induced interaction of β-arrestin with ADRB2; this behavior may be due to the light-induced interaction not causing a conformational change in β-arrestin that results in recruitment of MEK and ERK1/2. We consequently showed that the artificial induction of interactions between membrane receptors and their regulatory proteins via an optogenetic tool is a useful approach for investigating the intracellular trafficking of membrane receptors. Light-induced internalization of membrane receptors on cell surfaces and the manipulation of intracellular trafficking will be beneficial to unveiling significant roles of membrane receptors in living tissues and will also be useful for investigating the pathological processes of important diseases.

## Methods and Materials

### Materials and cDNA Construction

SNAP-Surface Alexa Fluor 647 and anti-SNAP-tag antibodies were obtained from New England Biolabs (USA). ISO and Dyngo4a were purchased from Tokyo Chemical Industry (Japan) and Abcam (USA), respectively. Anti-Rab5, anti-Rab7, anti-LAMP1, anti-ERK1/2 and anti-phosphorylated ERK1/2 antibodies were obtained from Cell Signaling Technology (USA). Anti-β2-adrenergic receptor (ADRB2) and anti-phosphorylated ADRB2 antibodies were purchased from Santa Cruz Biotechnology (USA). The SNAP-Surface Alexa Fluor 647 and Dyngo4a were solved in DMSO. The ISO was solved in H_2_O. The DNA fragments encoding SNAP-tag, ADRB2 and CIB were inserted between *Hind*III and *Xho*I sites in pcDNA3.1/V5-His (B) (Thermo Fisher Scientific, USA). The genes encoding CRY, mCherry and β-arrestin 2 were subcloned between *BamH*I and *Xba*I sites in pcDNA4/myc-His (B) (Thermo Fisher Scientific).

### Cell Cultivation

HEK293 cells were cultured in Dulbecco’s Modified Eagle Medium (D-MEM, Wako Pure Chemical Industry, Japan) containing 10% fetal bovine serum (FBS, Sigma-Aldrich, USA) and 1% penicillin/streptomycin (Gibco, Thermo Fisher Scientific, USA) under a 5% CO_2_ atmosphere at 37 °C. D-MEM supplemented with 10% FBS, 1% penicillin/streptomycin, 400 μg/ml G418 (Gibco) and 100 μg/ml zeocin (Thermo Fisher Scientific) was used for cultivating HEK293 cells stably expressing fusion proteins (HEK293_opt_ and HEK293_optCLIP_).

### Confocal Fluorescence Microscopy

Cells were cultured on 35-mm glass-bottomed dishes. To label the SNAP-tag, 0.5 μM SNAP-Surface Alexa Fluor 647 was added to the medium, and the cells were incubated for 30 min at 37 °C. After the cells were washed three times, the medium was exchanged by 2 ml of phenol red-free D-MEM containing HEPES, pH 7.4 (Wako Pure Chemical Industry). The cells were observed under a confocal microscope (IX-81, FV-1000D, Olympus). The images were acquired every 1 min. CRY activation was performed under a confocal microscope using a 20 mW 440-nm laser at 0.5% output with a scan speed of 2.0 μs/pixel. The fluorescence intensity of Arrestin_CRY_ was evaluated using the image analysis software FV10-ASW (Olympus). The number of ADRB2_CIB_ or Mdm2 fluorescence spots was counted using ImageJ. The diameter to detect the spots was set at 0.5–5 μm.

### Immunostaining of Early Endosomes, Late Endosomes and Lysosomes

HEK293_opt_ cells were cultured on 35-mm glass-bottomed dishes. ADRB2_CIB_ was labeled with SNAP-Surface Alexa Fluor 647 for 30 min and then washed three times with D-MEM. The cells were cultured in D-MEM and stimulated with blue light at an intensity of 3 mW/cm^2^ using an LED device (TH-211 × 200BL, Creating Customer Satisfaction, Japan) for the indicated times. The cells were fixed using 4% formaldehyde and then permeabilized with 0.2% Triton X-100. To avoid the non-specific adsorption of antibodies, the cells were treated with 0.2% gelatin from cold-water fish skin (Sigma-Aldrich) for 1 h at room temperature. Rab5, Rab7 and LAMP1 were labeled with the corresponding antibodies in PBS(+) overnight at 4 °C. After the cells were washed three times with PBS(+), they were stained with Alexa Fluor 488-conjugated goat anti-Rabbit IgG Secondary Antibody (Thermo Fisher Scientific) for 1 h at room temperature. The cells were washed three times with PBS(+) and then observed under a confocal microscope.

### Calculation of Manders’ Colocalization Coefficient

The extent of the colocalization of ADRB2_CIB_ with lysosomes was estimated using Manders’ colocalization coefficient.

$$\mathrm{Manders}\mbox{'}\,{\rm{colocalization}}\,{\rm{coefficient}}=\frac{{\sum }_{j}F{I}_{j{\rm{coloc}}}}{{\sum }_{i}F{I}_{i}}$$

where *FI*_*i*_ is the fluorescence intensity of Alexa Fluor 647 molecules attached to ADRB2_CIB_ in the pixel *i*, and *FI*_*j*coloc_ = 0 if the fluorescence intensity of Alexa 488, which is attached to LAMP1, in the pixel *j* is lower than a particular threshold, and *FI*_*j*coloc_ = *FI*_*j*_ if the fluorescence intensity of Alexa 488 in pixel *j* is equal to or larger than the threshold. The coefficient was calculated using the JACoP plug-in for ImageJ after applying a threshold of fluorescent intensity to each time-lapse image^42^. The thresholds were automatically determined based on the JACoP program.

### Quantification of ADRB2_CIB_ on the Cell Surface Using the ELISA Assay

HEK293_opt_ cells in 4-well plates were treated with 1.0 μM ISO or irradiated with blue light at 3 mW/cm^2^ for the indicated time. After the cells were fixed with 4% formaldehyde, they were treated with gelatin from cold-water fish skin for blocking. SNAP-tag moieties on the cell surface were labeled with an anti-SNAP-tag antibody for 60 min at room temperature. After the cells were washed twice with PBS(+), ECL anti-rabbit antibody linked to HRP was added to each well. The cells were incubated for 60 min at room temperature and then washed three times with PBS(+). After the addition of 350 μl of TMB solution (Thermo Fisher Scientific) and 30 min of incubation, 350 μl of 2 M sulfuric acid was added to each well. The OD_450_ value of 100 μl of solution was measured using a microplate reader (Bio-Rad, USA). The amount of ADRB2_CIB_ on the cell surface was defined by (OD − OD^mock^)/(OD^basal^ − OD^mock^) × 100, where OD^mock^ and OD^basal^ correspond to the OD of non-stimulated HEK293 cells and the OD from non-stimulated HEK293_opt_, respectively. To promote recycling of ADRB2_CIB_ in ISO-treated cells, cells were washed three times with 500 μl of acidic buffer (150 mM NaCl and 5 mM acetic acid) and then twice with 500 μl of PBS(+) to eliminate ISO.

### Detection of Ubiquitinated ADRB2_CIB_ Using Immunoprecipitation

HEK293_opt_ cells that had been cultured on a 4-well plate were stimulated with 3 mM/cm^2^ blue light using the LED device or 1.0 μM ISO for the indicated time. The cells were harvested using 200 μl of lysis buffer (50 mM HEPES (pH 7.5), 0.5% NP-40, 250 mM NaCl, 2 mM EDTA, 10% glycerol, 1 mM sodium orthovanadate, 1 mM sodium fluoride, 1 mM phenylmethylsulfonyl fluoride, 10 mg/ml leupeptin and MG-132 proteasome inhibitor). Anti-V5 antibody (Life Technologies) was added to each lysate, which was then gently shaken at 4 °C for 1 h. After protein G Sepharose (GE Healthcare, USA) was added, the lysate was shaken at 4 °C for 1 h. The precipitate was washed three times with 1 ml of lysis buffer and dissolved in 100 μl of sample buffer (125 mM Tris, pH 6.8, 10% glycerol, 4% SDS, 0.006% bromophenol blue and 10% mercaptoethanol). The samples were heated at 65 °C for 10 min and then subjected to SDS-PAGE using 10% acrylamide gels. The proteins were transferred onto nitrocellulose membranes (Bio-Rad, USA). To block the non-specific binding of antibodies, the membrane was treated with 1% skim milk in Tris-buffered saline for 1 h. ADRB2 and ubiquitin were blotted using their corresponding antibodies. The blotted bands were detected using an image analyzer (ImageQuant LAS-4000, GE Healthcare), and the intensity of each band was measured with an ImageQuant TL software.

### Statistical Analysis

Statistical significance was determined using unpaired Student’s t-tests (two-tailed) and Bonferroni post-hoc test. Differences with *P* values < 0.05 were considered statistically significant.

## Electronic supplementary material

Movie S1a

Movie S1b

Movie S1c

Movie S2a

Movie S2b

Movie S3a

Movie S3b

Supplementary information
