# Supplementary material for: Unique Roles of β-Arrestin in GPCR Trafficking Revealed by Photoinducible Dimerizers
Source: Sci Rep. 2018 Jan 12;8:677. doi: 10.1038/s41598-017-19130-y (PMC5766490; doi:10.1038/s41598-017-19130-y)
Supplement: Supplementary file 8 — Supplementary information [file 41598_2017_19130_MOESM8_ESM.pdf]

Supporting Information

Unique Roles of β-Arrestin in GPCR Trafficking Revealed by Photoinducible Dimerizers

Osamu Takenouchi†, Hideaki Yoshimura† and Takeaki Ozawa†

† Department of Chemistry, School of Science, The University of Tokyo

*Correspondence should be addressed to Takeaki Ozawa

Email: ozawa@chem.s.u-tokyo.ac.jp

Table of contents

1. **Light-induced endocytosis in the transfected HEK293 cells S-2**
2. **Quantification of the number of $\mathrm{ADRB2_{CIB}}$ fluorescence spots S-4**
3. **Characterization of light-induced endocytosis of $\mathrm{ADRB2_{CIB}}$ S-6**
4. **Observation of $\mathrm{ADRB2_{CIB}}$ and endosome marker proteins after light stimulation. S-7**
5. **Temporal changes of colocalization of $\mathrm{Arrestin_{CRY}}$ with ADRB2 after stopping irradiation S-9**
6. **Investigation of the ability of $\mathrm{Arrestin_{CRY}}$ to desensitize ISO-stimulated ADRB2 .... S-10**
7. **Examination of a state of $\mathrm{ADRB2_{CIB}}$ during light stimulation S-11**
8. **Detection of phosphorylated ERK1/2 and $\mathrm{ADRB2_{CIB}}$ after light stimulation S-12**
9. **Applicability to other membrane receptors S-13**
10. **Full blots images S-15**
11. **Supplemental information of the movies S-16**

1. Light-induced endocytosis in the transfected HEK293 cells

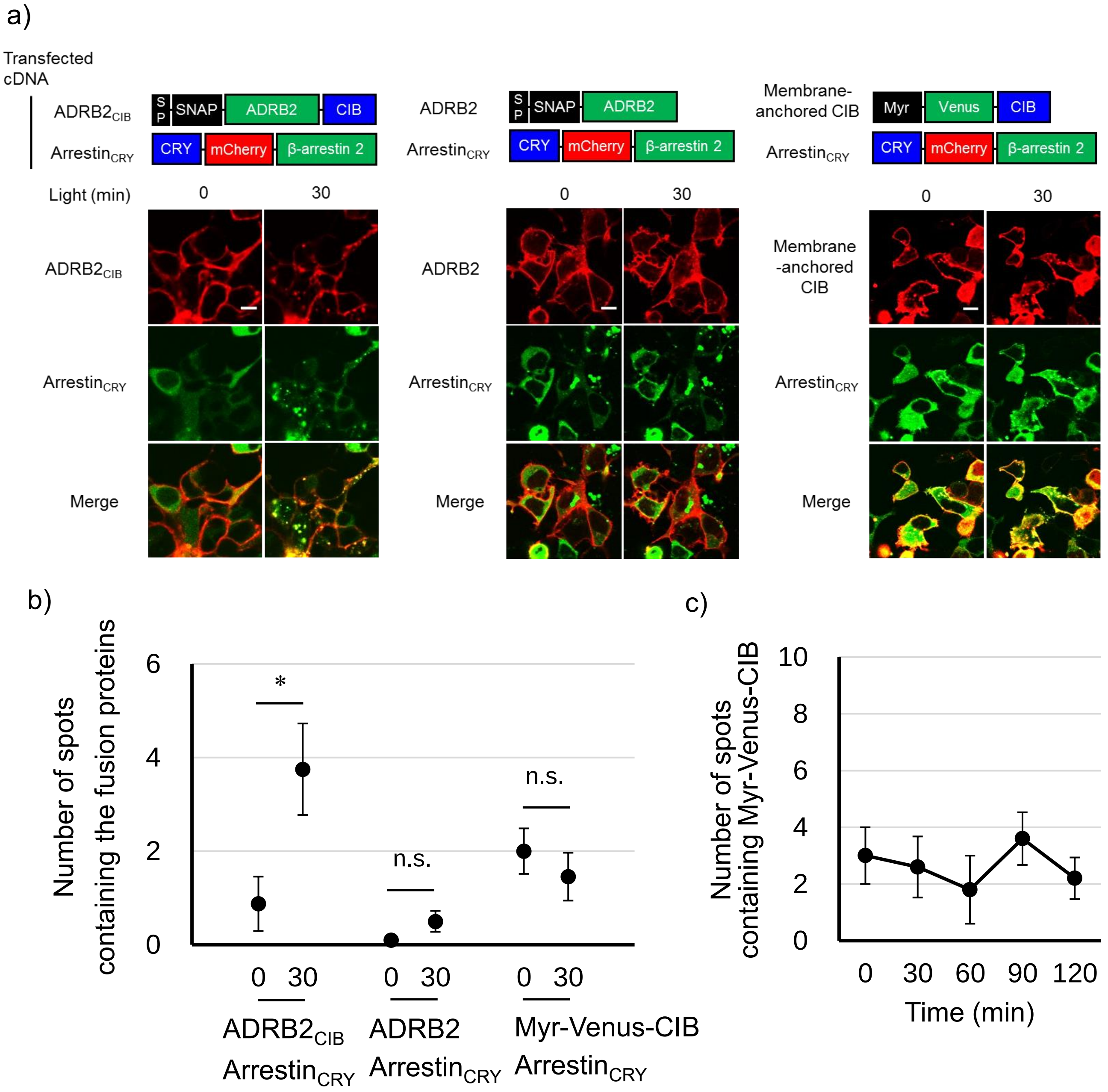

Supplementary Figure 1 | Light-induced endocytosis in the transfected HEK293 cells. a) Images of fluorescence from HEK293 cells transfected with cDNAs encoding $ADRB2_{CIB}$ and $Arrestin_{CRY}$ (left), SNAP-ADRB2 (ADRB2) and $Arrestin_{CRY}$ (middle), or membrane-anchored CIB (Myr-Venus-CIB) and $Arrestin_{CRY}$ (right). The cells were irradiated with blue light for 30 min under a confocal microscope. Red, $ADRB2_{CIB}$(left), ADRB2 (middle), Myr-Venus-CIB (right); green, $Arrestin_{CRY}$. Bar shows 10 µm. b) Temporal changes of the numbers of spots containing the fusion proteins per cell. The

number of spots in HEK293 cells expressing $Arrestin_{CRY}$ and $ADRB2_{CIB}$, ADRB2, or Myr-Venus-CIB were counted. Bar: mean ± s.e.m. (n = 8, 10, 11 cells, respectively). Statistical analysis was performed with paired Student's t-test (Two tailed). $*P < 0.05$ ($P = 0.016$). c) Temporal changes in the number of spots containing Myr-Venus-CIB during 120 min irradiation. (n = 5 cells)

2. Quantification of the number of $ADRB2_{CIB}$ fluorescence spots and the colocalization

a)

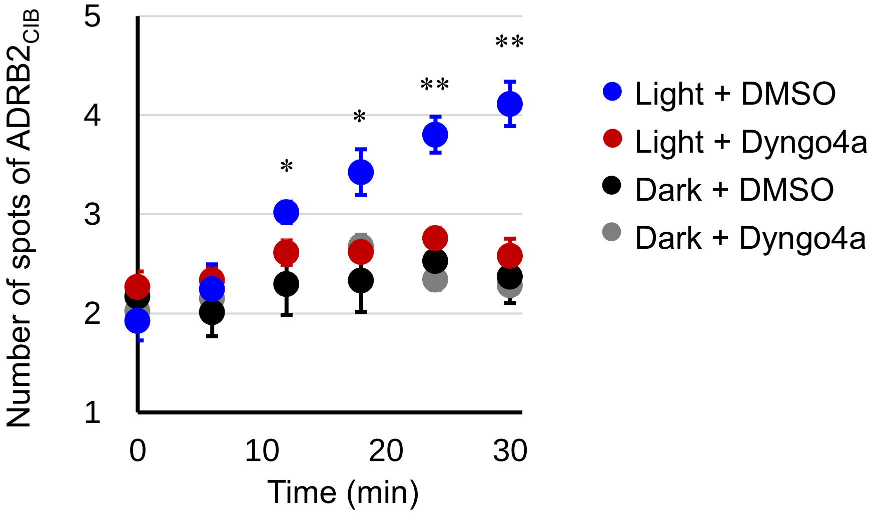

b)

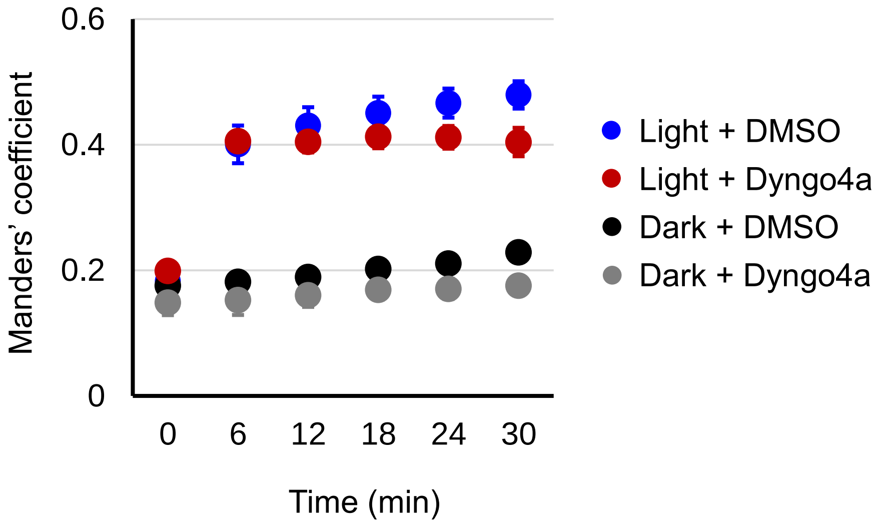

Supplementary Figure 2 | Quantification of the number of $ADRB2_{CIB}$ fluorescence spots.

Time courses of a) the number of $ADRB2_{CIB}$ fluorescence spots and b) the colocalization of $Arrestin_{CRY}$ with $ADRB2_{CIB}$ in $HEK293_{opt}$ cells as a function of blue light irradiation and 30 μM Dingo4a treatment. The number of spots per cell was calculated by normalizing total number of fluorescence spots in each imaging with the number of nucleus stained using Hoechst 33342. The final concentration of DMSO was 0.1%. Blue:

light irradiated and no Dyngo 4a; red: irradiated and Dyngo 4a present; black: non-irradiated and no Dyngo 4a; gray: non-irradiated and Dyngo 4a present. Bar: mean ± s.e.m. (n = 4 from four individual experiments). Statistical significance was determined using a two-way ANOVA (v.s. Light + Dyngo 4a). * *P* values < 0.05 (12 min; $P = 0.047$, 18 min; $P = 0.018$), ***P* values < 0.01 (24 min; $P = 0.0021$, 30 min; $P = 0.0013$).

3. Characterization of light-induced endocytosis of ADRB2$_{CIB}$

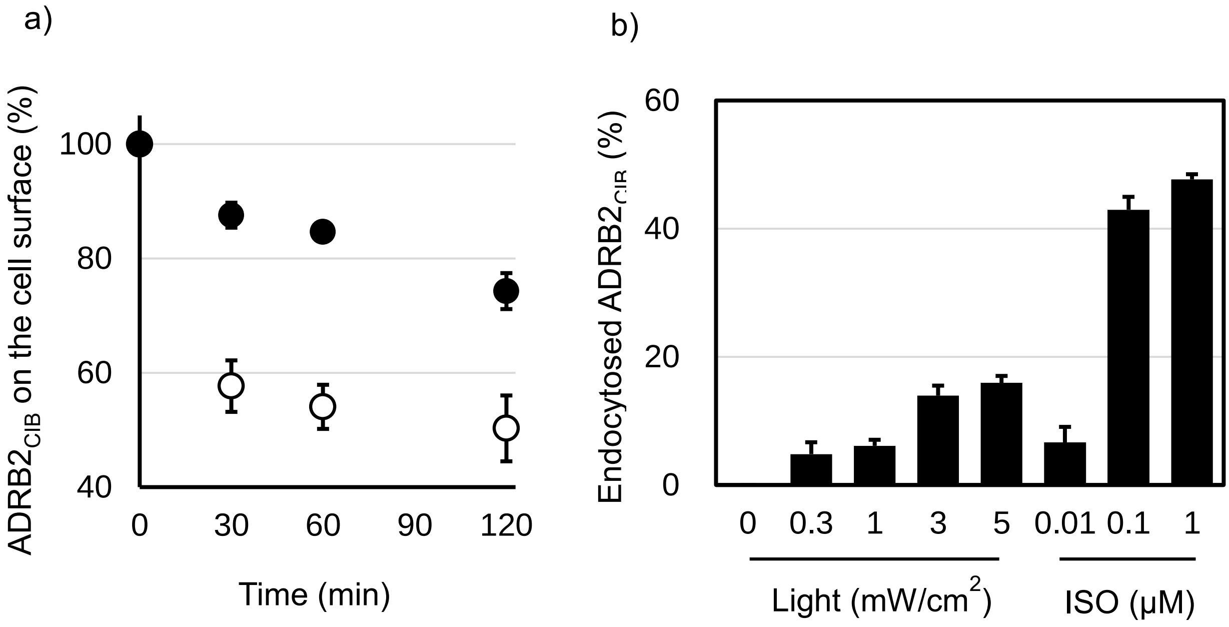

Supplementary Figure 3 | Characterization of light-induced endocytosis of ADRB2$_{CIB}$.

a) Time courses of ADRB2$_{CIB}$ on the cell surface of HEK293$_{opt}$ cells stimulated with light at an intensity of 3 mW/cm^2 (filled circles) and 1.0 µM ISO (open circles) for the indicated times. After the cells were fixated, the amount of ADRB2$_{CIB}$ on the cell surface was quantified using an ELISA. b) Light intensity dependency of endocytosis of ADRB2$_{CIB}$ in HEK293$_{opt}$ cells stimulated with blue light at various intensities or with 0.01, 0.1 and 1.0 µM ISO for 60 min. The amount of endocytosed ADRB2$_{CIB}$ was quantified using an ELISA. Bar: mean ± s.e.m. (n = 8 from two individual experiments).

4. Observation of the ADRB2CIB and endosome marker proteins after light stimulation.

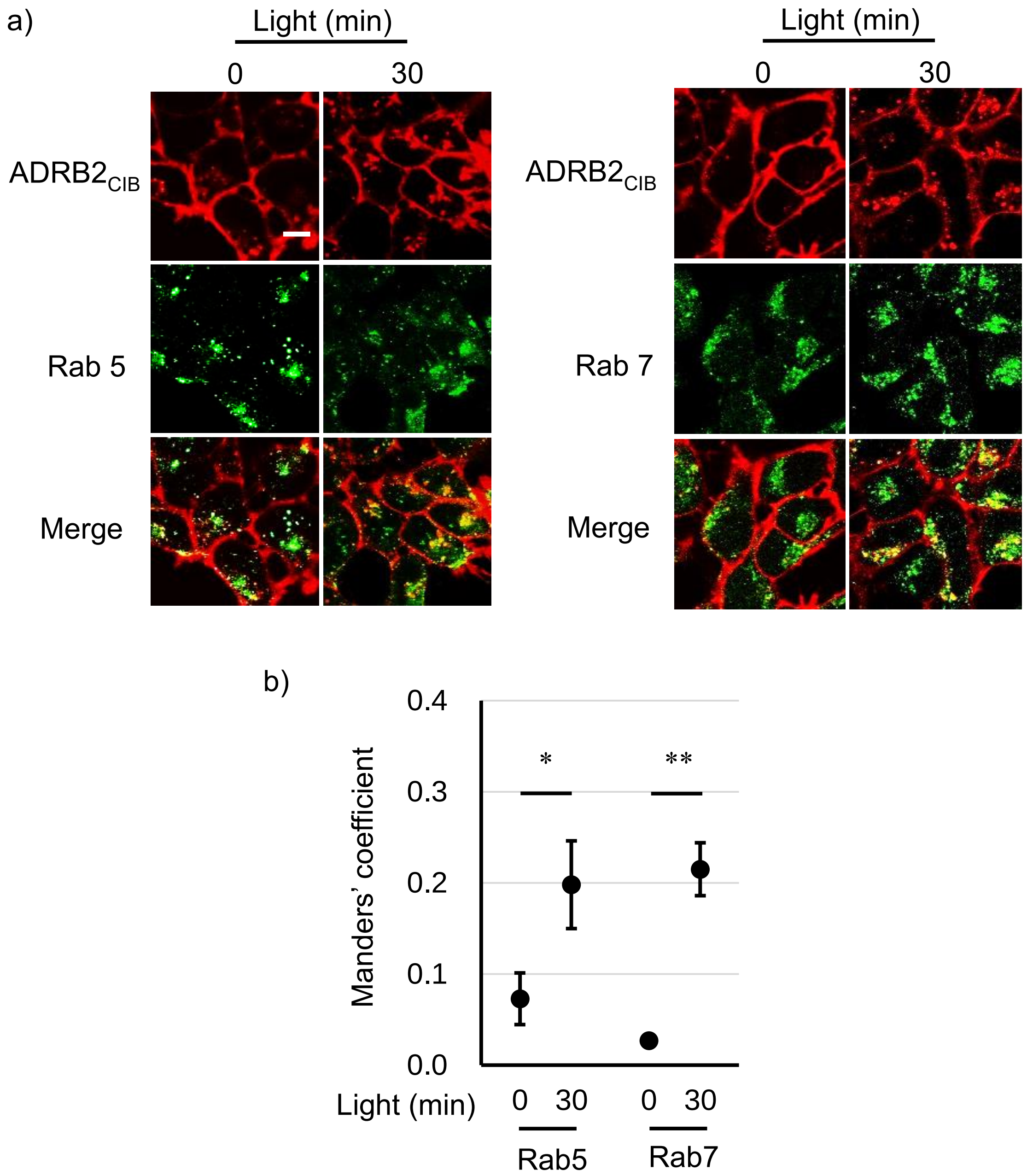

Supplementary Figure 4 | Observation of ADRB2CIB and endosome marker proteins after light stimulation.

a) Images of ADRB2CIB and endosome marker protein fluorescence in $HEK293_{opt}$ cells before and after blue light illumination at an intensity of 3 mW/cm^2 for 30 min. After the cells were fixed, Rab5 or Rab7 was immunostained with its specific antibody. Red,

$ADRB2_{CIB}$; green, Rab5 or Rab7. Bar represents 10 μm. b) Quantification of colocalization of $ADRB2_{CIB}$ with Rab5 and Rab7. Bars: mean ± s.e.m, (n = 3). Statistical analysis was performed with unpaired t-test (two tailed), $*P < 0.05$ ($P = 0.018$), $**P < 0.01$($P = 0.00039$).

5. Temporal changes in the colocalization of Arrestin$_{CRY}$ with ADRB2 after stopping irradiation

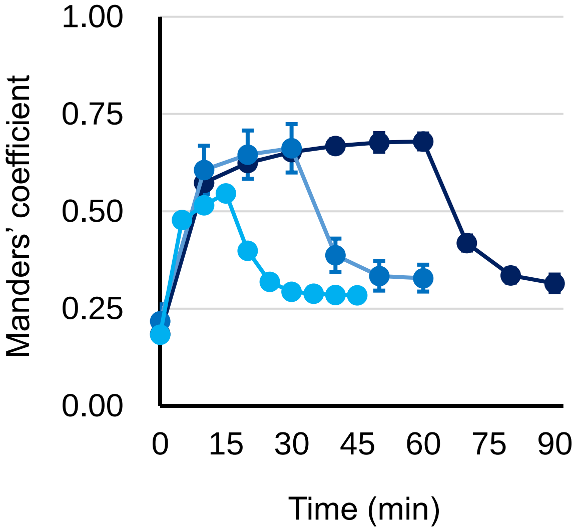

Supplementary Figure 5 | Temporal changes in the colocalization of Arrestin$_{CRY}$ with ADRB2 after stopping irradiation.

Time courses in the colocalization of Arrestin$_{CRY}$ with ADRB2$_{CIB}$ after stopping irradiation. The cells were stimulated with blue light for 15 min (pale blue), 30 min (deep blue), and 60 min (black) and then incubated in the dark. Bars: mean ± s.e.m (n = 3 from three individual experiments).

6. Investigation of the ability of Arrestin$_{CRY}$ to desensitize ISO-activated ADRB2.

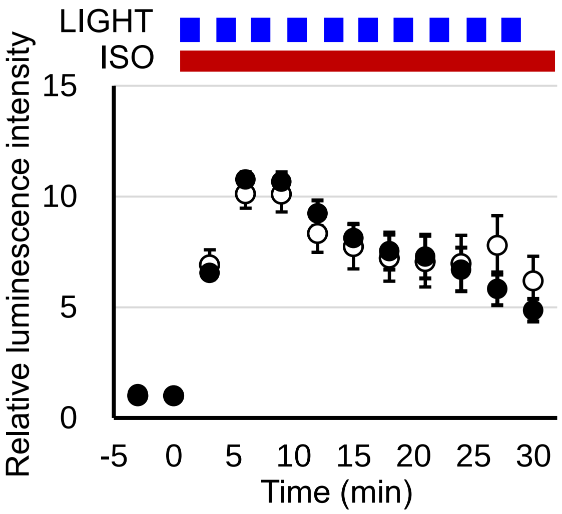

Supplementary Figure 6 | Investigation of the ability of Arrestin$_{CRY}$ to desensitize ISO-activated ADRB2$_{CIB}$.

Temporal changes in the luminescence intensity from GloSensor upon stimulation of ISO under light irradiation. The HEK293$_{opt}$ cells expressing the GloSensor were pretreated with 30 μM Dyngo4a and exposed to 1.0 μM ISO in the presence or absence of light irradiation. The luminescent intensity from the GloSensor was measured at every 3 mins. Blue light irradiation was performed for 1 min during an interval of each measurement. The luminescence intensities at each time point were normalized against those at 0 min. Filled circle; the irradiated cells, open circle; non-irradiated cells. Bars: mean ± s.e.m, (n = 7 from two individual experiments).

7. Examination of a state of ADRB2CIB during light irradiation

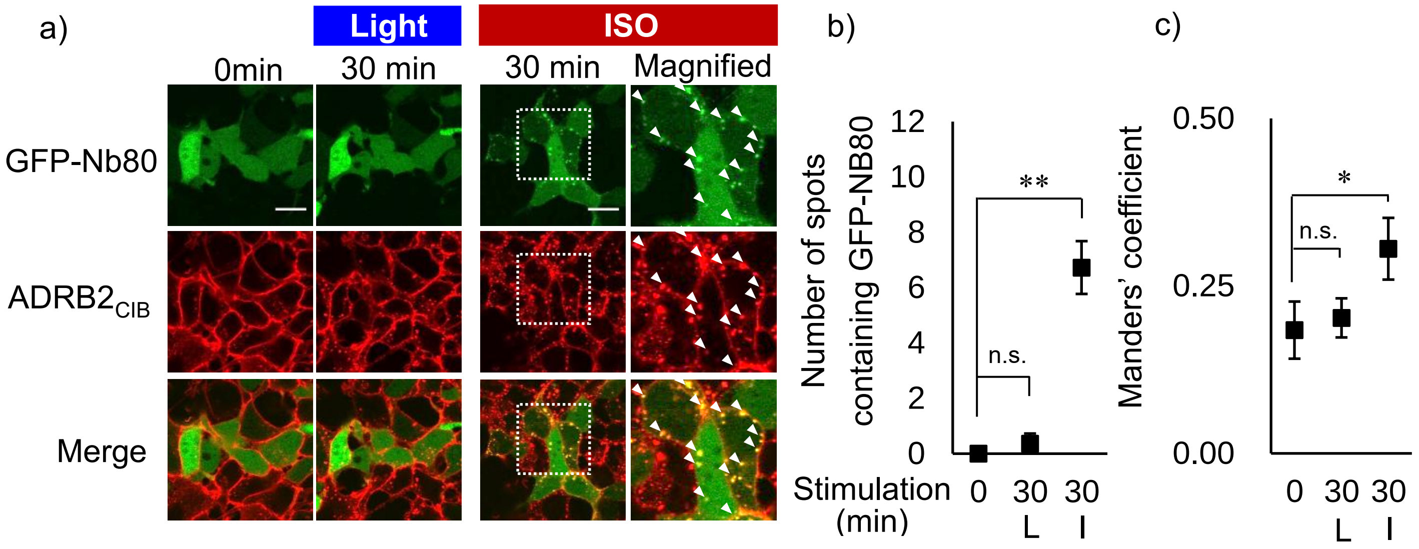

Supplementary Figure 7 | Examination of a state of ADRB2CIB during Light Irradiation.

The HEK293opt cells expressing GFP-fused Nb80 were stimulated with light or 1.0 µM ISO. Temporal changes of a) fluorescent imaging of GFP-Nb80 and ADRB2CIB, b) number of particles containing GFP-Nb80 per cell, c) Manders' colocalization coefficient of Nb80 with ADRB2CIB in the HEK293opt cells stimulated with light or ISO. Scale bar shows 20 µm. Error bars: mean ± s.e.m, (n = 11 cells). L; Light, I; ISO. Statistical analysis was performed with Bonferroni post-hoc, $^{**}P < 0.01$ ($P = 8.8\times10^{-5}$) * $P < 0.05$ (P = 0.018), n.s. $P > 0.05$ (b; $P = 0.68$, c; $P = 0.48$).

8. Detection of Phosphorylated ERK1/2 and ADRB2CIB after light stimulation

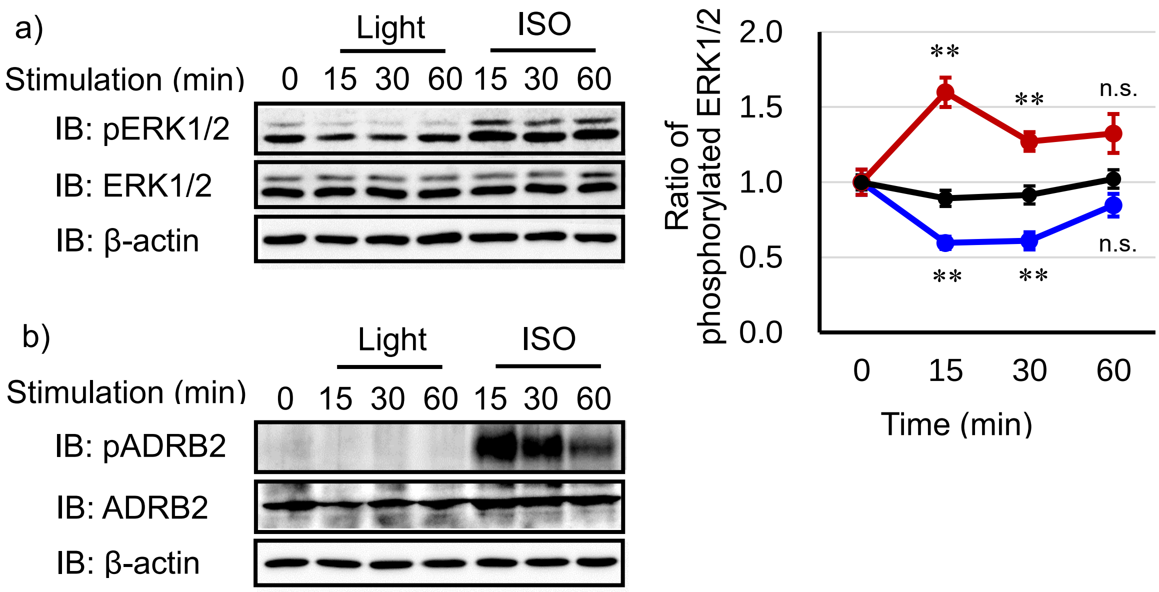

Supplementary Figure 8 | Detection of Phosphorylated ADRB2CIB and ERK1/2 after Light Irradiation.

Western blotting analysis for the detection of phosphorylated a) ERK1/2 and b) ADRB2CIB after stimulation with blue light (3 mW/cm^2) or 1.0 μM ISO. HEK293$_{opt}$ cells were starved in D-MEM containing 0.1% FBS for 12 h before the stimulation. Phosphorylation of ERK1/2 is quantified by the ratio of phosphorylated ERK1/2 to total ERK1/2 quantities, which were determined from the Western blots. The ratios were normalized by the values at 0 min. Blue: light irradiated; red: ISO stimulated; black: non-irradiated. Bar: mean ± s.e.m (n=8 from two individual experiments). Statistical analysis was performed by Bonferroni post-hoc test (non-irradiated v.s. light-irradiated or ISO-stimulated): $^{**}P$ values < 0.01 (Light: $P = 1.8\times10^{-3}$ (15 min), 7.4×10^{-3} (30 min); ISO: $P = 5.6\times10^{-5}$ (15 min), 3.1×10^{-3} (30 min)), n.s. $P > 0.05$ (a; Light: 0.28, ISO: 0.16).

9. Applicability to other membrane receptors

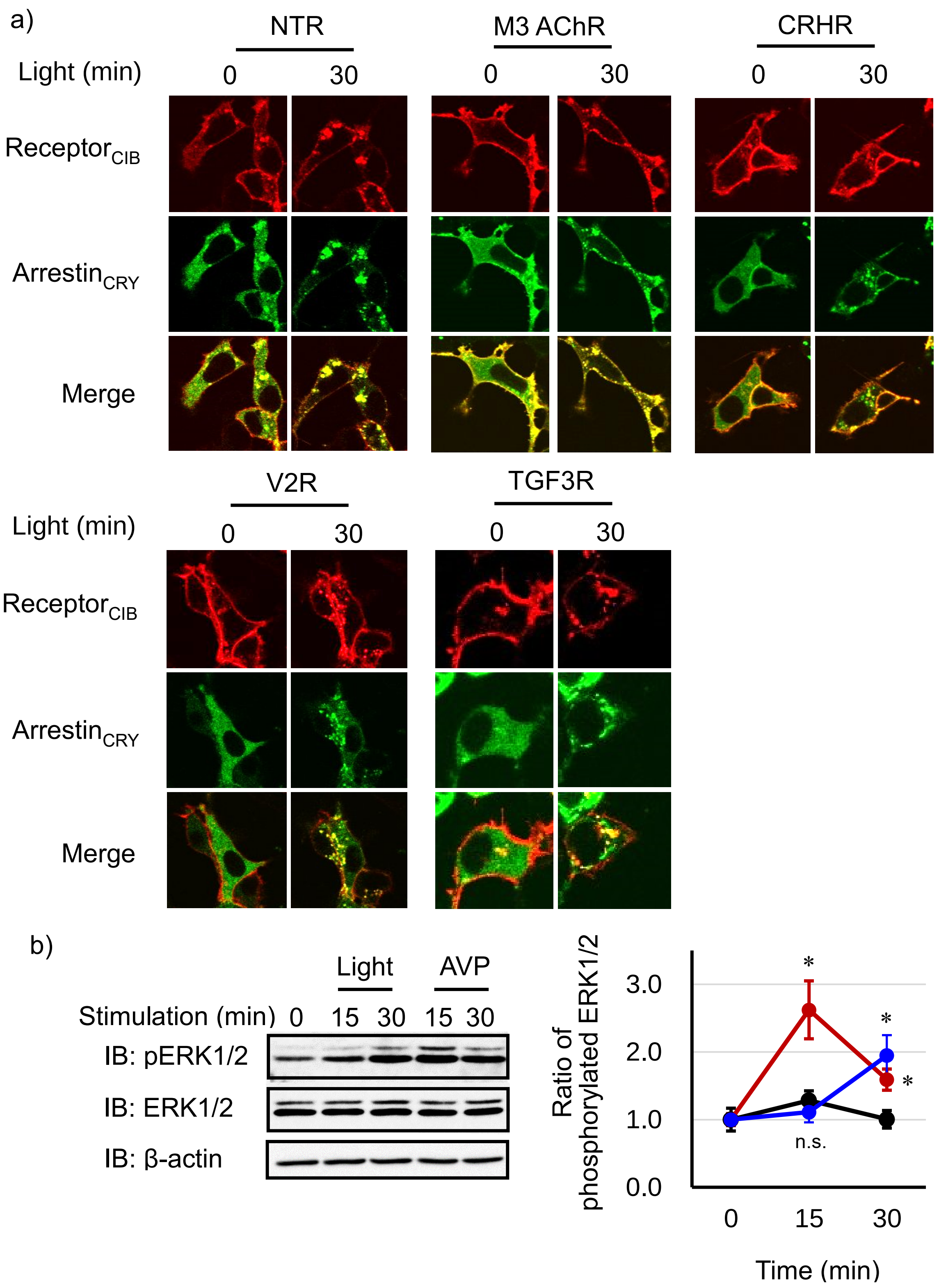

Supplementary Figure 9 | Applicability of other membrane receptors.

a) Images of CIB-fused receptor and Arrestin$_{CRY}$ fluorescence in HEK293 cells stimulated with blue light. HEK293 cells were transfected with cDNA encoding Arrestin$_{CRY}$ and CIB-fused membrane receptors. Neurotensin receptor, NTR; muscarinic acetylcholine receptor M3, M3 mAChR; corticotropin releasing-factor receptor, CRHR; vasopressin 2 receptor, V2R; and transforming growth factor 3 receptor, TGF3R. b) Temporal changes of ERK1/2 phosphorylation after light-induced interaction of Arrestin$_{CRY}$ with V2R$_{CIB}$. HEK293 cells expressing Arrestin$_{CRY}$ and V2R$_{CIB}$ were starved in D-MEM containing 0.1% FBS for 12 h. The cells were stimulated with blue light (3 mW/cm^2) or 1.0 μM vasopressin (AVP). Phosphorylation of ERK1/2 was quantified by the ratio of phosphorylated ERK1/2 to the total ERK1/2 quantities. The ratios were normalized by the values at 0 min. Blue: light irradiated; red: AVP stimulated; black: non-irradiated. Bar: mean ± s.e.m (n = 8 from two individual experiments). Statistical analysis was performed by Bonferroni post-hoc test (non-irradiated v.s. light-irradiated or AVP-stimulated): **P* values < 0.05 (Light: *P* =, 0.024 (30 min); AVP: *P* = 0.020 (15 min), 0.024 (30 min)), n.s. *P* > 0.05 (Light: 0.82).

10. Full blots images.

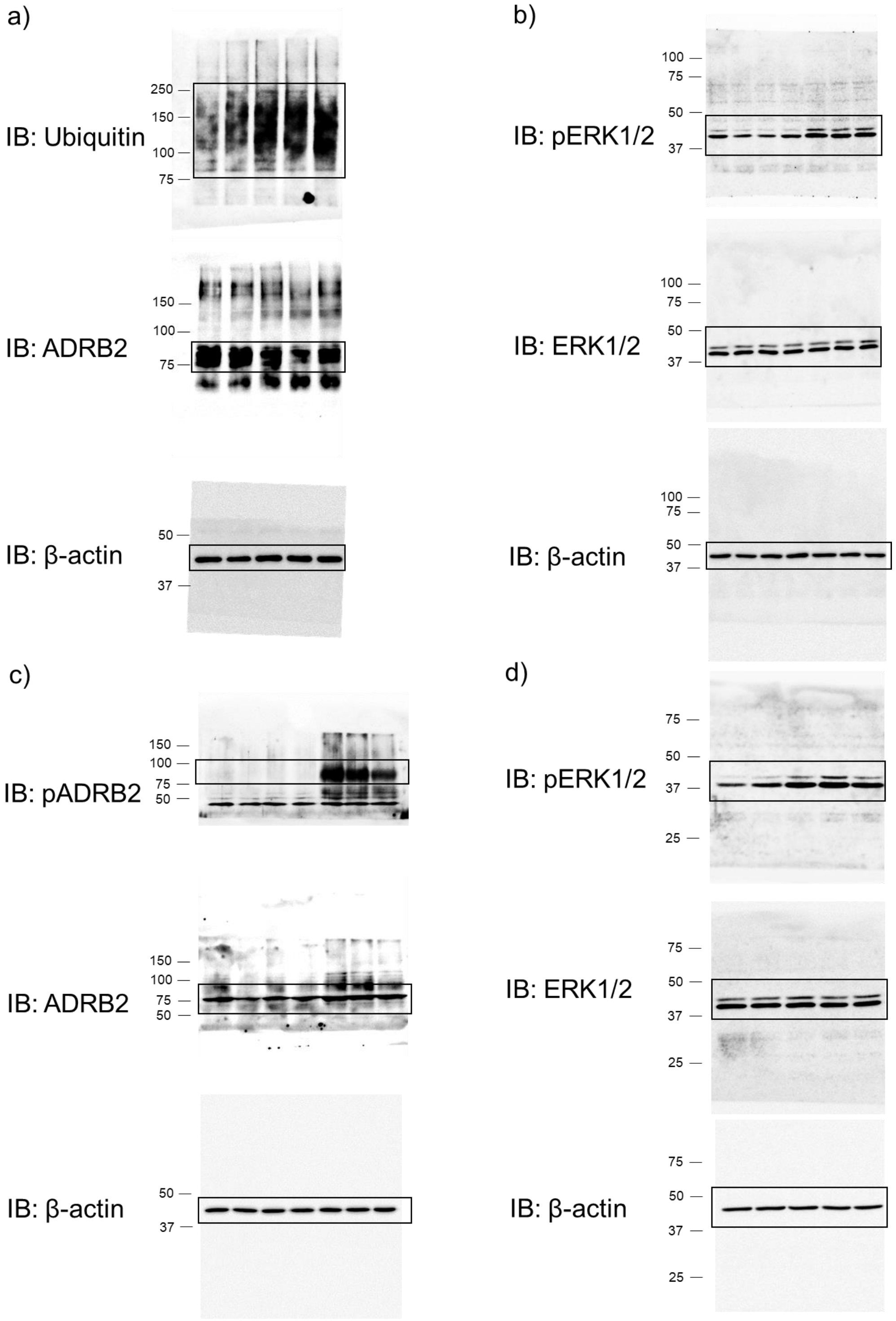

Supplementary Figure 10 | Full blots images.

Full blots images of (a) Fig. 4c, (b) Supplementary Fig. 8a, (c) Supplementary Fig. 8b, (d) Supplementary Fig. 9b

11. Supplemental information of the movies

Movie S1a-c: Light-induced endocytosis of ADRB2$_{CIB}$.

Time-laps movies of the HEK293$_{opt}$ cell. The cells were stimulated with 440-nm laser light for 30 min. The movies showed the dynamics of a) ADRB2$_{CIB}$, b) Arrestin$_{CRY}$, and c) both of the ADRB2$_{CIB}$ (red) and Arrestin$_{CRY}$ (green). Scale bar, 20 μm.

Movie S2a, b: Redistribution of Arrestin$_{CRY}$ in the cytosol under the dark condition.

Time-laps movies of the HEK293$_{opt}$ cell. The movies demonstrated the dynamics of a) Arrestin$_{CRY}$ (green) and b) ADRB2$_{CIB}$ (red). Scale bar, 20 μm.

Movie S3a, b: Observation of the dynamics of Mdm2.

Time-laps movies of the HEK293$_{optCLIP}$ cell expressing mCherry-fused Mdm2. a) The cells were irradiated for 60 min. b) the cells were stimulated for 30 min and incubated for 30 min without irradiation of blue light. Scale bar, 10 μm.
